# Methyl 3-(2-hy­droxy­benzyl­idene)-2-methyl­dithio­carbazate

**DOI:** 10.1107/S160053681201731X

**Published:** 2012-04-25

**Authors:** S. K. S. Hazari, Sebastian Suarez, Biplab Ganguly, Fabio Doctorovich, Tapashi G. Roy

**Affiliations:** a University of Chittagong, Chittagong 4331, Bangladesh; b Departamento de Química Inorgánica, Analítica y Química, Física/INQUIMAE–CONICET, Facultad de Ciencias Exactas y Naturales, Universidad de Buenos Aires, Argentina

## Abstract

In the title compound, C_10_H_12_N_2_OS_2_, the thione and S-methyl groups are *syn*. An intra­molecular bifurcated O—H⋯(S,N) hydrogen bond occurs.

## Related literature

For the biological activity of sulfur-ligand compounds, see: French & Blang (1965 ▶); Ali & Livingstone (1974 ▶); Ali *et al.* (1995 ▶); Hazari *et al.* (1999 ▶, 2002 ▶). For the synthesis and characterization of sulfur–nitro­gen-containing ligands, see: Hazari *et al.* (2002 ▶, 2006 ▶). For a related structure, see: Hazari *et al.* (2012 ▶).
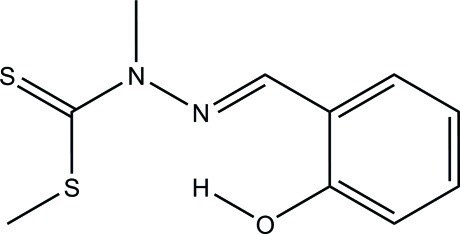


## Experimental

### 

#### Crystal data

C_10_H_12_N_2_OS_2_
*M* *_r_* = 240.34
Monoclinic, 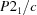

*a* = 11.3561 (16) Å
*b* = 8.9033 (13) Å
*c* = 11.5045 (16) Å
β = 91.411 (13)°
*V* = 1162.8 (3) Å^3^
*Z* = 4
Mo *K*α radiation
μ = 0.43 mm^−1^
*T* = 298 K
0.35 × 0.30 × 0.22 mm

#### Data collection

Oxford Diffraction Gemini CCD S Ultra diffractometer
Absorption correction: multi-scan (*CrysAlis PRO*; Oxford Diffraction, 2009 ▶) *T*_min_ = 0.859, *T*_max_ = 0.917
16680 measured reflections
2833 independent reflections
2039 reflections with *I* > 2σ(*I*)
*R*_int_ = 0.052

#### Refinement

*R*[*F* ^2^ > 2σ(*F* ^2^)] = 0.053
*wR*(*F* ^2^) = 0.171
*S* = 1.05
2833 reflections
144 parameters
H atoms treated by a mixture of independent and constrained refinement
Δρ_max_ = 0.42 e Å^−3^
Δρ_min_ = −0.41 e Å^−3^

### 

Data collection: *CrysAlis PRO* (Oxford Diffraction, 2009 ▶); cell refinement: *CrysAlis PRO*; data reduction: *CrysAlis PRO*; program(s) used to solve structure: *SHELXS86* (Sheldrick, 2008 ▶); program(s) used to refine structure: *SHELXL97* (Sheldrick, 2008 ▶); molecular graphics: *ORTEP-3 for Windows* (Farrugia, 1999 ▶); software used to prepare material for publication: *WinGX* (Farrugia, 1999 ▶).

## Supplementary Material

Crystal structure: contains datablock(s) global, I. DOI: 10.1107/S160053681201731X/qm2064sup1.cif

Structure factors: contains datablock(s) I. DOI: 10.1107/S160053681201731X/qm2064Isup2.hkl

Supplementary material file. DOI: 10.1107/S160053681201731X/qm2064Isup3.cml

Additional supplementary materials:  crystallographic information; 3D view; checkCIF report

## Figures and Tables

**Table 1:** Hydrogen-bond geometry (Å, °)

*D*—H⋯*A*	*D*—H	H⋯*A*	*D*⋯*A*	*D*—H⋯*A*
O1—H1*O*⋯S2	0.91 (5)	2.67 (5)	3.453 (2)	145 (4)
O1—H1*O*⋯N1	0.91 (5)	1.88 (5)	2.678 (3)	145 (4)

**Fig. 1.**
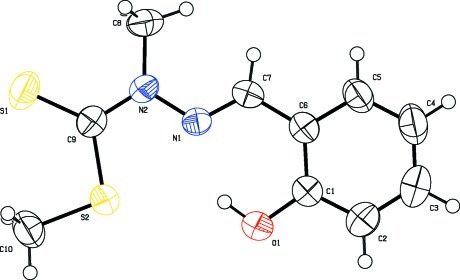
The molecular structure of (I) showing the atom-labelling scheme and displacement ellipsoids at the 50% probability level.

**Fig. 2.**
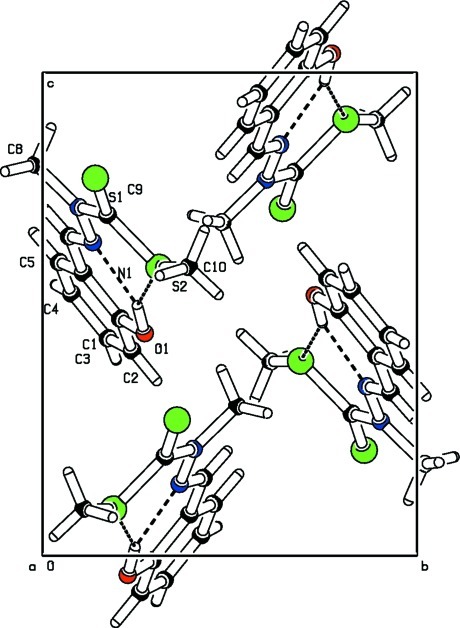
Cristal packing for the title compound viewed along c.

## supplementary crystallographic information

### Comment

It is well known that sulfur-containing compounds have potential biological activities (French & Blang, 1965; Ali & Livingstone, 1974; Ali *et al.*, 1995; Hazari *et al.*, 1999; Hazari *et al.*, 2002). As a continuation of the synthesis and characterization of sulfur-nitrogen containing ligands (Hazari *et al.*, 1999; Hazari *et al.*, 2002) and their metal complexes, the present investigation is an attempt to prepare a complex of vanadium(IV) with the Schiff base ligand (*L*, prepared by the condensation of salicylaldehyde and *N*-methyl-S– methyldithiocarbamate). Although a greenish yellow complex was obtained, during crystallization from ethanol, the ligand (m.p. 126–128°C) was regenerated in the form of a crystal. Hence, the crystal structure of the ligand has been described.

In the crystal structure of (1) (Fig.1), there is a bifurcated hydrogen bond involving O1 - H10 ··· S2 and O1 - H10 ··· N1 interactions. The O1 S2 and O1 N1 distances are 3.453 (2) Å and 2.678 (3) Å respectively.

### Experimental

The title compound was isolated by following four steps synthetic procedure:

Step1. Synthesis of *N*-methyl-*S*-methyldithiocarbamate: Potassium hydroxide (11.5 g) was dissolved in 60 ml of 90% ethanol and the mixture was cooled down to 273 K in an ice bath. To this, methylhydrazine (11.1 ml) was added slowly with mechanical stirring. A solution of carbondisulfide (12 ml) was added dropwise from a burette with constant stirring over a period of an hour. During the addition of carbondisulfide, the temperature of the reaction mixture was not allowed to rise above 279 K. A yellow colored solution was obtained. After adding carbondisulfide, methyl iodide (12.5 ml) was added from a burette dropwise with vigorous mechanical stirring. After the complete addition, the mixture was stirred for further 15 minutes, whereupon well formed shining crystals appeared. The product was separated by filtration and washed with water and recrystallized from ethanol and dried in a vacuum desiccator over silica gel. Yield: 15.25 g, *M*.pt.: 361–363 K.

Step 2. Synthesis of methyl-*N*-(2-hydroxybenzyledine)-*N*-methyl hydrazinecarbodithionate, *L* (1): A hot solution of salicyladehyde (1.04 ml, 10 mmol) in absolute ethanol (40 ml) was mixed with hot solution of *N*-methyl-S– methyldithiocarbamate (1.36 g, 10 mmol) in the same solvent. The mixture was refluxed for 6 h. on a water bath. After reducing the volume, a yellowish white product appeared which was filtered off. This product was washed with ethanol several times (3 *x* 2 ml) and dried in a vacuum desiccator over silica gel. Yield: 1.65 g. *M*.pt.: 399–401 K.

Step 3. Attempted preparation of the oxovanadium(IV) complex with (I): Vanadyl acetylacetonate [VO2(acac)2] (2.65 g, 10 mmol) was dissolved in dry ethanol, in which a hot solution of *L* (2.4 g, 10 mmol) in dry ethanol was added. The mixture was refluxed for 6 h. on water bath. After reducing the volume and standing over night a light greenish yellow product appeared, which was washed with ethanol for several times and dried in a vacuum desiccator over silica gel. Melting point of product was 443–445 K.

Step 4. Crystallization: The product was dissolved in ethanol to which half volume of petroleum ether was added (10/5 ml *v*/*v*). The solution was left for several days after which the title compound, (I), was deposited as crystals.

### Refinement

Methyl groups were idealized (C—H = 0.96 A °) and allowed to ride. In all cases, H-atom displacement parameters were taken as *U*_iso_(H) = 1.5Ueq(C) for methyl groups or 1.2Ueq(C,O) otherwise.

### Crystal data

**Table:**

C_10_H_12_N_2_OS_2_	*F*(000) = 504
*M**_r_* = 240.34	*D*_x_ = 1.373 Mg m^−^^3^
Monoclinic, *P*2_1_/*c*	Mo *K*α radiation, λ = 0.71073 Å
Hall symbol: -P 2ybc	Cell parameters from 0 reflections
*a* = 11.3561 (16) Å	θ = 4.0–29.1°
*b* = 8.9033 (13) Å	µ = 0.43 mm^−^^1^
*c* = 11.5045 (16) Å	*T* = 298 K
β = 91.411 (13)°	Prism, colourless
*V* = 1162.8 (3) Å^3^	0.35 × 0.30 × 0.22 mm
*Z* = 4	

### Data collection

**Table:**

Oxford Diffraction Gemini CCD S Ultra diffractometer	2039 reflections with *I* > 2σ(*I*)
Graphite monochromator	*R*_int_ = 0.052
ω scans	θ_max_ = 29.1°, θ_min_ = 4.0°
Absorption correction: multi-scan (*CrysAlis PRO*; Oxford Diffraction, 2009)	*h* = −14→15
*T*_min_ = 0.859, *T*_max_ = 0.917	*k* = −11→11
16680 measured reflections	*l* = −14→15
2833 independent reflections	

### Refinement

**Table:**

Refinement on *F*^2^	Primary atom site location: structure-invariant direct methods
Least-squares matrix: full	Secondary atom site location: difference Fourier map
*R*[*F*^2^ > 2σ(*F*^2^)] = 0.053	Hydrogen site location: inferred from neighbouring sites
*wR*(*F*^2^) = 0.171	H atoms treated by a mixture of independent and constrained refinement
*S* = 1.05	*w* = 1/[σ^2^(*F*_o_^2^) + (0.0801*P*)^2^ + 0.6976*P*] where *P* = (*F*_o_^2^ + 2*F*_c_^2^)/3
2833 reflections	(Δ/σ)_max_ < 0.001
144 parameters	Δρ_max_ = 0.42 e Å^−^^3^
0 restraints	Δρ_min_ = −0.41 e Å^−^^3^

### Special details

**Table:**

Geometry. All e.s.d.'s (except the e.s.d. in the dihedral angle between two l.s. planes) are estimated using the full covariance matrix. The cell e.s.d.'s are taken into account individually in the estimation of e.s.d.'s in distances, angles and torsion angles; correlations between e.s.d.'s in cell parameters are only used when they are defined by crystal symmetry. An approximate (isotropic) treatment of cell e.s.d.'s is used for estimating e.s.d.'s involving l.s. planes.
Refinement. Refinement of *F*^2^ against ALL reflections. The weighted *R*-factor *wR* and goodness of fit *S* are based on *F*^2^, conventional *R*-factors *R* are based on *F*, with *F* set to zero for negative *F*^2^. The threshold expression of *F*^2^ > σ(*F*^2^) is used only for calculating *R*-factors(gt) *etc*. and is not relevant to the choice of reflections for refinement. *R*-factors based on *F*^2^ are statistically about twice as large as those based on *F*, and *R*- factors based on ALL data will be even larger.

### Fractional atomic coordinates and isotropic or equivalent isotropic displacement parameters (Å^2^)

**Table:**

	*x*	*y*	*z*	*U*_iso_*/*U*_eq_	
S2	0.74513 (6)	0.31081 (8)	0.59687 (6)	0.0526 (2)	
S1	0.88032 (7)	0.14078 (11)	0.78107 (8)	0.0705 (3)	
O1	0.47228 (19)	0.2727 (3)	0.4609 (2)	0.0610 (6)	
N1	0.56325 (18)	0.1339 (2)	0.64913 (17)	0.0411 (5)	
N2	0.65614 (19)	0.0963 (2)	0.72163 (18)	0.0447 (5)	
C6	0.3631 (2)	0.1080 (3)	0.5859 (2)	0.0434 (5)	
C7	0.4625 (2)	0.0705 (3)	0.6613 (2)	0.0437 (6)	
C1	0.3700 (2)	0.2072 (3)	0.4911 (2)	0.0444 (6)	
C8	0.6446 (3)	−0.0200 (3)	0.8097 (2)	0.0582 (7)	
H8A	0.5658	−0.0592	0.8067	0.087*	
H8B	0.6609	0.0221	0.8852	0.087*	
H8C	0.6994	−0.0995	0.7952	0.087*	
C5	0.2542 (2)	0.0437 (4)	0.6081 (3)	0.0591 (7)	
H5	0.2485	−0.024	0.6692	0.071*	
C9	0.7587 (2)	0.1735 (3)	0.7057 (2)	0.0449 (6)	
C2	0.2700 (3)	0.2389 (4)	0.4249 (3)	0.0578 (7)	
H2	0.2745	0.3047	0.3624	0.069*	
C3	0.1633 (3)	0.1742 (4)	0.4505 (3)	0.0681 (9)	
H3	0.0966	0.1964	0.4052	0.082*	
C10	0.8904 (3)	0.3907 (4)	0.5977 (3)	0.0668 (8)	
H10A	0.8935	0.469	0.5405	0.1*	
H10B	0.9466	0.3141	0.5798	0.1*	
H10C	0.9087	0.4316	0.6732	0.1*	
C4	0.1553 (3)	0.0767 (4)	0.5430 (3)	0.0725 (9)	
H4	0.0833	0.0339	0.561	0.087*	
H7	0.444 (3)	0.007 (4)	0.717 (3)	0.082 (11)*	
H1O	0.527 (4)	0.253 (5)	0.518 (4)	0.087 (12)*	

### Atomic displacement parameters (Å^2^)

**Table:**

	*U*^11^	*U*^22^	*U*^33^	*U*^12^	*U*^13^	*U*^23^
S2	0.0478 (4)	0.0548 (4)	0.0548 (4)	−0.0099 (3)	−0.0043 (3)	0.0076 (3)
S1	0.0517 (5)	0.0886 (6)	0.0702 (5)	0.0089 (4)	−0.0151 (4)	0.0073 (4)
O1	0.0498 (12)	0.0718 (13)	0.0614 (12)	−0.0079 (10)	0.0019 (10)	0.0231 (11)
N1	0.0439 (11)	0.0376 (10)	0.0420 (10)	0.0021 (8)	0.0009 (8)	0.0019 (8)
N2	0.0446 (11)	0.0443 (11)	0.0449 (11)	0.0052 (9)	−0.0033 (9)	0.0064 (9)
C6	0.0461 (13)	0.0399 (12)	0.0444 (12)	−0.0027 (10)	0.0045 (10)	−0.0078 (10)
C7	0.0509 (14)	0.0382 (12)	0.0424 (13)	−0.0036 (10)	0.0073 (10)	0.0003 (10)
C1	0.0461 (14)	0.0414 (12)	0.0458 (13)	0.0007 (10)	0.0044 (10)	−0.0043 (10)
C8	0.0687 (19)	0.0524 (15)	0.0535 (15)	0.0052 (13)	0.0018 (13)	0.0158 (12)
C5	0.0512 (16)	0.0632 (18)	0.0629 (17)	−0.0167 (13)	0.0031 (13)	0.0018 (14)
C9	0.0455 (14)	0.0455 (13)	0.0438 (13)	0.0070 (10)	0.0016 (10)	−0.0048 (10)
C2	0.0597 (17)	0.0575 (16)	0.0558 (16)	0.0069 (13)	−0.0035 (13)	−0.0027 (13)
C3	0.0525 (18)	0.079 (2)	0.072 (2)	0.0066 (15)	−0.0142 (15)	−0.0106 (17)
C10	0.0519 (17)	0.076 (2)	0.073 (2)	−0.0139 (15)	0.0075 (15)	0.0017 (16)
C4	0.0472 (17)	0.088 (2)	0.083 (2)	−0.0162 (16)	−0.0050 (15)	−0.0018 (19)

### Geometric parameters (Å, º)

**Table:**

S2—C9	1.754 (3)	C8—H8A	0.96
S2—C10	1.796 (3)	C8—H8B	0.96
S1—C9	1.639 (3)	C8—H8C	0.96
O1—C1	1.353 (3)	C5—C4	1.366 (5)
O1—H1O	0.91 (4)	C5—H5	0.93
N1—C7	1.286 (3)	C2—C3	1.380 (5)
N1—N2	1.370 (3)	C2—H2	0.93
N2—C9	1.368 (3)	C3—C4	1.378 (5)
N2—C8	1.457 (3)	C3—H3	0.93
C6—C5	1.392 (4)	C10—H10A	0.96
C6—C1	1.408 (4)	C10—H10B	0.96
C6—C7	1.446 (4)	C10—H10C	0.96
C7—H7	0.89 (4)	C4—H4	0.93
C1—C2	1.381 (4)		
			
C9—S2—C10	102.00 (15)	C4—C5—C6	122.2 (3)
C1—O1—H1O	108 (3)	C4—C5—H5	118.9
C7—N1—N2	120.0 (2)	C6—C5—H5	118.9
C9—N2—N1	116.2 (2)	N2—C9—S1	123.3 (2)
C9—N2—C8	122.8 (2)	N2—C9—S2	112.69 (19)
N1—N2—C8	121.0 (2)	S1—C9—S2	124.01 (17)
C5—C6—C1	117.8 (3)	C3—C2—C1	120.8 (3)
C5—C6—C7	118.7 (2)	C3—C2—H2	119.6
C1—C6—C7	123.5 (2)	C1—C2—H2	119.6
N1—C7—C6	121.1 (2)	C4—C3—C2	120.2 (3)
N1—C7—H7	126 (2)	C4—C3—H3	119.9
C6—C7—H7	113 (2)	C2—C3—H3	119.9
O1—C1—C2	118.1 (3)	S2—C10—H10A	109.5
O1—C1—C6	122.3 (2)	S2—C10—H10B	109.5
C2—C1—C6	119.7 (3)	H10A—C10—H10B	109.5
N2—C8—H8A	109.5	S2—C10—H10C	109.5
N2—C8—H8B	109.5	H10A—C10—H10C	109.5
H8A—C8—H8B	109.5	H10B—C10—H10C	109.5
N2—C8—H8C	109.5	C5—C4—C3	119.4 (3)
H8A—C8—H8C	109.5	C5—C4—H4	120.3
H8B—C8—H8C	109.5	C3—C4—H4	120.3

### Hydrogen-bond geometry (Å, º)

**Table:**

*D*—H···*A*	*D*—H	H···*A*	*D*···*A*	*D*—H···*A*
O1—H1*O*···S2	0.91 (5)	2.67 (5)	3.453 (2)	145 (4)
O1—H1*O*···N1	0.91 (5)	1.88 (5)	2.678 (3)	145 (4)
